# Prognostic value and early response stratification of a multi-biomarker panel in cervical cancer patients undergoing chemoradiotherapy

**DOI:** 10.3389/fonc.2025.1686716

**Published:** 2025-10-30

**Authors:** Yu Wang, Na Gan, Shan Ning, Yinting Qiu

**Affiliations:** Department of Gynecology, Pingxiang Maternal and Child Health Hospital, Pingxiang, Jiangxi, China

**Keywords:** cervical cancer, chemoradiotherapy, multi-biomarker panel, treatment response, prognosis, human papillomavirus, inflammation, risk stratification

## Abstract

**Aims:**

Despite progress in chemoradiotherapy (CRT), outcomes in cervical cancer still vary widely. Minimally invasive biomarkers may enable risk stratification and treatment optimization.

**Methods:**

We prospectively enrolled 164 International Federation of Gynecology and Obstetrics (FIGO) IB–IVA patients, all receiving CRT plus brachytherapy. Baseline blood markers and HPV subtypes were assessed. Treatment response was evaluated at three months, and progression-free (PFS) and overall survival (OS) were measured over a median of 36 months.

**Results:**

Elevated squamous cell carcinoma antigen (SCC-Ag), Cancer antigen 125 (CA125), Interleukin-6 (IL-6), C-reactive protein (CRP), and high neutrophil-to-lymphocyte ratio and platelet-to-lymphocyte ratio (NLR/PLR) correlated with advanced disease. At three months, 87.1% showed complete response (CR) or partial response (PR). Higher IL-6, CRP, SCC-Ag, CA125, and NLR/PLR were linked to poorer response. At 36 months, PFS and OS were 65.2% and 74.5%, respectively. High-risk patients had lower PFS (58.1% *vs*. 72.4%) and OS (64.5% *vs*. 82.0%), independent of stage, with no increase in severe toxicity.

**Conclusions:**

A multi-biomarker panel shows superior discrimination for early response and is prognostic for survival in locally advanced cervical cancer. Larger, multi-institutional studies are warranted to validate this panel, standardize assays, and investigate additional markers or imaging-based strategies, ultimately facilitating more personalized therapy and improved outcomes. shows superior discrimination for early response and is prognostic for survival in locally advanced cervical cancer.

## Introduction

Cervical cancer remains a critical global health challenge, with more than 80% of cases and deaths occurring in low- and middle-income countries—especially in regions like Sub-Saharan Africa and South-Eastern Asia, where incidence rates are notably high due to limited healthcare access and preventive services (1, 2). This worldwide prevalence is compounded by socioeconomic disparities, leading to disproportionately high burdens in underserved populations and lower-income areas (3). The current standard of care for cervical cancer is chemoradiotherapy (CRT), which includes external beam radiation combined with concurrent chemotherapy, followed by brachytherapy. Across historical cohorts, radiographic response rates after definitive CRT decline with advancing FIGO stage, with consistently higher CR/PR frequencies in stage IB–II than in stage III–IVA, although absolute rates vary by imaging modality, timing, and cohort mix (4, 5). Although this regimen is largely effective, its use is complicated by variability in treatment response and significant recurrence risks (6).

In response, a range of prognostic and predictive biomarkers—ranging from tumor markers such as SCC-Ag and CA125, to molecular markers including HPV subtypes, to immune markers like IL-6—has been investigated to enhance treatment outcomes. However, the predictive accuracy of individual biomarkers often varies across different populations and disease stages (7).

A multi-biomarker strategy has been proposed to address the biological complexity underlying tumor progression (8, 9). By integrating various pathways, composite panels may offer improved predictive power over individual markers (10, 11). Clinically, there is a growing need for reliable, noninvasive tools that can accurately forecast treatment response and identify high-risk patients early, thus facilitating more aggressive or alternative therapeutic interventions (8, 9). Translational research opportunities abound for validating these biomarker panels in real-world settings and closing the gap between laboratory findings and clinical implementation, ultimately improving patient outcomes (11, 12).

In this study, we evaluated whether a multi-biomarker panel can accurately predict short-term therapeutic response among cervical cancer patients undergoing chemoradiotherapy, and we also examined the panel’s ability to predict long-term outcomes such as progression-free and overall survival. Our findings hold substantial clinical promise, potentially informing treatment decisions, guiding follow-up intervals, and identifying candidates for adjunctive therapies to mitigate poor prognoses.

## Methods

This was a prospective, single-center cohort study conducted at Pingxiang Maternal and Child Health Hospital, between March 2019 to May 2024. The study protocol was approved by the Ethics Committee of Pingxiang Maternal and Child Health Hospital (Approval number: PY-2018-30). Written informed consent was obtained from all participants before enrollment, and all procedures were performed in accordance with the Declaration of Helsinki and local regulations.

Inclusion Criteria: 1), Histologically confirmed cervical cancer (FIGO stages IB–IVA); 2), Planned for definitive CRT (external beam radiotherapy + concurrent chemotherapy, followed by brachytherapy); 3), Age ≥18 years; 4), Adequate hematologic, renal, and hepatic function (assessed by routine blood tests); 5), Willingness to provide blood samples and undergo required follow-up.

Exclusion Criteria: 1), Prior radiation or chemotherapy for cervical cancer; 2), Presence of metastatic disease (FIGO stage IVB) or other malignancies; 3), Significant comorbidities contraindicating CRT (e.g., severe cardiac or hepatic dysfunction); 4), Pregnancy or breastfeeding at enrollment; 5), Inability to attend regular follow-up visits or comply with study procedures.

A total of 164 patients were assessed for eligibility. Thirteen were excluded due to incomplete baseline data or loss to follow-up, leaving 151 patients in the final analysis.

### Treatment protocol

Radiotherapy: All patients received external beam radiotherapy (EBRT) to the pelvis with a total dose of approximately 45–50.4 Gy in 25–28 fractions over 5–6 weeks. Image-guided or conventional brachytherapy was administered after EBRT, delivering an additional 28–30 Gy to the high-risk clinical target volume.

Concurrent Chemotherapy: Weekly intravenous cisplatin (40 mg/m^2^) was administered concurrently with EBRT in most cases, unless contraindicated. Alternative regimens (e.g., carboplatin) were considered for patients with renal dysfunction or other cisplatin contraindications.

### Post-treatment management

In our study, patients who did not achieve a CR at three months—classified as partial response, stable disease, or progressive disease—were evaluated for additional interventions in consultation with the multidisciplinary tumor board. For PR, if residual disease was deemed resectable and the patient’s performance status was acceptable, a surgical approach was considered. In select cases, additional chemotherapy regimens (platinum-based with or without a targeted agent) were administered to patients with persistent lesions, provided no contraindications existed. Patients presenting with SD or PD often underwent further imaging to confirm disease extent and evaluate potential metastasis. Those with localized residual disease were offered salvage radiotherapy or re-irradiation if deemed feasible, whereas patients with advanced or metastatic progression received palliative chemotherapy or best supportive care.

### Biomarker assessment

Peripheral blood samples (5–10 mL) were drawn at baseline (within 1 week before starting CRT). SCC-Ag and CA125 were measured using standard immunoassays. Inflammatory and Immunologic Markers, CRP and IL-6 were quantified via enzyme-linked immunosorbent assay. Complete blood count (CBC) differentials were performed to derive the NLR, PLR, and lymphocyte-to-monocyte ratio (LMR). Serum LDH levels were measured using an automated enzymatic assay. High-risk HPV detection and genotyping (e.g., HPV-16, HPV-18, others) were performed using polymerase chain reaction (PCR)-based assays.

All tests were performed in the hospital clinical laboratory using validated, commercial platforms. SCC−Ag and CA125 were quantified by automated immunoassay and are reported in ng/mL and U/mL, respectively. Our laboratory reference intervals are SCC−Ag <2.0 ng/mL and CA125 <35 U/mL, which we prespecified as analysis cut−offs. IL−6 and CRP were measured by ELISA−based immunoassays and reported in pg/mL and mg/L. Our laboratory reference intervals are IL−6 <6.0 pg/mL and CRP <5.0 mg/L, used as prespecified cut−offs. Complete blood counts were obtained on automated hematology analyzers. Derived ratios used standard definitions (NLR = absolute neutrophils/lymphocytes; PLR = platelets/lymphocytes; LMR = lymphocytes/monocytes). We prespecified thresholds NLR >3.0, PLR >180, and LMR <3.0 from laboratory practice and prior literature. LDH was measured enzymatically (U/L) with >245 U/L prespecified from the laboratory reference interval (120–245 U/L).

### Response evaluation

Short-Term Response: Three months after completing CRT, computed tomography (CT) scans and a clinical examination were performed to assess tumor response. Treatment response was defined according to the Response Evaluation Criteria in Solid Tumors (RECIST) 1.1: complete response (CR), partial response (PR), stable disease (SD), or progressive disease (PD).

Long-Term Follow-Up: Patients were followed at 3–4-month intervals for the first 2 years, followed by 6-month intervals thereafter, for a total of 3 years post-treatment follow-up. PFS was calculated from the date of treatment initiation to the date of documented disease progression or death from any cause, whichever occurred first. OS was calculated from the date of treatment initiation to death from any cause, or the last follow-up date if still alive.

Reading conditions and modality rationale: Three−month response per RECIST 1.1 was determined from CT plus clinical examination by institutional radiologists as part of routine care; no central imaging review was performed. Radiologists were not provided the research biomarker dataset and were unaware of the composite score definition; RECIST calls were based on imaging and clinical findings only. We prespecified CT as the primary response modality to ensure a uniform, pragmatic assessment across the entire cohort in line with local standard−of−care and access. MRI or PET−CT could be obtained if clinically indicated, but were not used to define the primary RECIST endpoint in order to avoid inter−modality variability in this single−center study.

Three months after CRT, response was assessed by contrast−enhanced CT plus clinical examination using RECIST 1.1: complete response (CR) = disappearance of all target lesions and normalization of any pathological lymph nodes (short axis <10 mm); partial response (PR) = ≥30% decrease in the sum of diameters (SoD) of target lesions from baseline; progressive disease (PD) = ≥20% increase in SoD (with an absolute increase ≥5 mm) or the appearance of new lesion(s) or unequivocal progression of non−target disease; stable disease (SD) = neither PR nor PD criteria met. RECIST calls were based on imaging and clinical findings only, as detailed above.

### Follow−up and surveillance

Patients were reviewed every 3–4 months for the first 2 years and every 6 months thereafter to 36 months. At each visit, clinicians obtained interval history (symptoms including pain, bleeding, discharge, urinary/bowel changes, weight loss) and performed a pelvic examination (speculum inspection of the cervix/vaginal vault and bimanual/rectovaginal assessment of parametria and nodal basins). For patients with an intact cervix after definitive CRT, cervicovaginal cytology (Pap) was obtained at ~12 months and then annually. Reflex hrHPV testing or earlier repeat cytology was performed at the clinician’s discretion for abnormal results. Beyond the protocolized 3−month CT used for RECIST response, subsequent imaging was performed when clinically indicated (new symptoms or abnormal examination) or for routine surveillance at the treating physician’s discretion. The preferred modalities were contrast−enhanced pelvic CT or MRI for local/pelvic concerns and chest CT for thoracic symptoms or equivocal findings. PET−CT was reserved to clarify equivocal imaging or staging prior to salvage therapy. When feasible, biopsy of suspicious mucosal, nodal, or metastatic lesions was obtained to confirm recurrence. Progression during follow−up was defined by RECIST 1.1 on imaging for measurable disease, histologic confirmation of recurrence, or unequivocal radiographic progression adjudicated in multidisciplinary review when histology was not possible.

### Composite biomarker panel construction

Biomarkers were included based on clinical relevance and significant associations (p < 0.1) in univariate analyses. For each biomarker, patients received 1 point if the value exceeded a predefined risk cutoff, or 0 points otherwise. A composite score was generated, then dichotomized into “high-risk” *vs*. “low-risk” groups using either the median split or an optimal cut point determined by ROC analysis.

For the four prespecified panel markers—SCC−Ag (>2.0 ng/mL), CA125 (>35 U/mL), IL−6 (≥6.0 pg/mL), and NLR (>3.0)—cut−offs were prespecified from our laboratory reference intervals and widely used literature values; no individual marker cut−off was tuned on this cohort. Each marker above its cut−off was assigned 1 point (otherwise 0), and points were summed (0–4). The only data−driven step was selecting the composite score threshold by ROC/Youden using 3−month CR/PR ***vs*** SD/PD as the criterion, which yielded High−risk = score ≥2 and Low−risk = score 0–1. CRP/PLR/LMR/LDH were evaluated as individual comparators but are not part of the final panel. For each patient, assign 1 point if a marker exceeds its cut−off (otherwise 0), then sum points (range 0–4). Risk groups were Low−risk = score 0–1 and High−risk = score ≥2, with the threshold selected by ROC/Youden using 3−month CR/PR ***vs*** SD/PD as the outcome. CRP and PLR were analyzed as individual comparators but are not included in the final composite score.

### Toxicity assessment

Treatment−related adverse events were prospectively recorded at weekly on−treatment visits and at follow−up. Grades were assigned per CTCAE v5.0. To distinguish early and delayed effects, toxicities were classified as acute (from the start of EBRT through 90 days after completion of all radiotherapy [EBRT + brachytherapy]) and late (>90 days after radiotherapy completion to last follow−up). For summary tables, we counted the worst grade per patient within each system category (hematologic, gastrointestinal, genitourinary, other) and time window to avoid double−counting.

### Statistical analysis

All statistical analyses were conducted using R (Version 4.4), with two-sided p < 0.05 considered significant. Baseline characteristics were summarized as means ± standard deviations or medians (ranges) for continuous variables and as frequencies (percentages) for categorical variables. Between-group comparisons (e.g., high- *vs*. low-biomarker subsets or responders *vs*. non-responders) employed the Chi-square or Fisher’s exact test for categorical data and Student’s t test or the Mann-Whitney U test for continuous data, depending on distribution. ROC curves and area under the curve (AUC) metrics were used to evaluate the discriminative performance of individual biomarkers and the composite biomarker panel for short-term treatment response. Kaplan-Meier estimates characterized PFS and OS, and differences in survival curves were assessed by the log-rank test. Multivariable Cox proportional hazards models, adjusted for key prognostic factors such as FIGO stage, tumor size, and lymph node status, were fitted to determine whether the composite biomarker score independently predicted PFS and OS. *Sample Size Considerations*: This study was powered based on preliminary data suggesting a moderate effect size for certain biomarkers. We aimed for at least 150 evaluable patients to detect a minimum hazard ratio of ~1.5 for high-risk *vs*. low-risk biomarker status, assuming 80% power and a two-sided alpha of 0.05.

*Continuous modeling and internal check*: In sensitivity analyses, the four panel biomarkers were also modeled as continuous variables to avoid information loss (SCC−Ag, CA125, and IL−6 were log2−transformed; NLR was modeled per 1−unit increase). For short−term response (CR/PR *vs*. SD/PD), we fit a logistic regression using these continuous markers and reported AUC (with DeLong comparisons *vs*. the dichotomized panel). For survival, we fit Cox models with the same continuous markers and reported Harrell’s C−index. To assess internal stability, we implemented a chronological split at the cohort’s median enrollment date (early = derivation; late = validation). The score definition (≥2 *vs*. 0–1) and the continuous−marker model coefficients were frozen from the derivation set and applied to the validation set without re−tuning; we summarized discrimination (AUC/C−index) and prognostic separation (log−rank and Cox HR) in the validation set.

*Model parsimony and events*: To limit over−fitting, the primary multivariable Cox models for PFS and OS included four prespecified variables: FIGO stage (III/IVA *vs* I/II), tumor size (≥4 cm *vs <*4 cm), lymph−node metastasis (positive *vs* negative), and the composite biomarker score (≥2 *vs* 0–1). We report observed event counts and events−per−variable (EPV) for each endpoint. Treatment−delivery variables (overall treatment time, cumulative HRCTV EQD2, cisplatin cycles) were assessed in separate sensitivity models by adding each variable to the base model; we did not fit a single model containing all covariates simultaneously. Discrimination was summarized with Harrell’s C−index.

*Treatment delivery by risk group*: We summarized HR−CTV EQD2 (Gy), overall treatment time (days), and cisplatin cycles by composite risk group (score ≥2 *vs* 0–1) as median [IQR] and as guideline−aligned thresholds (EQD2 ≥85 Gy; OTT >56 days; cycles ≥4). Between−group differences were tested using the Mann–Whitney U test for continuous variables and χ²/Fisher’s exact tests for categorical thresholds. Treatment−delivery variables were kept visible in multivariable analyses and, in sensitivity analyses, were added one at a time to the base clinical model.

## Results

**Figure 1:**
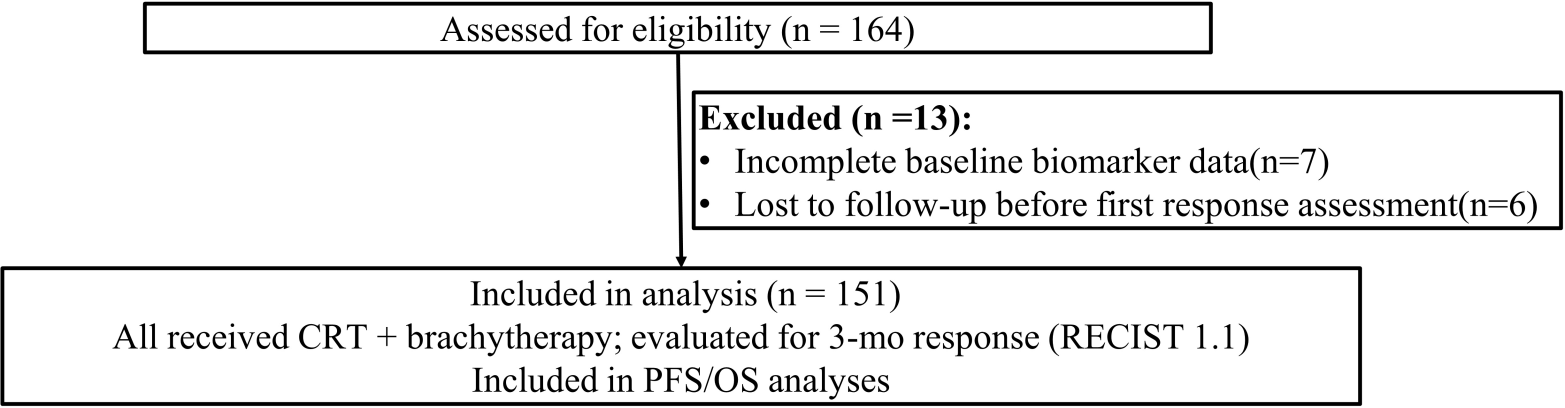
Study cohort flow diagram. Assessed for eligibility (n = 164); excluded (n = 13) due to incomplete baseline biomarker data (n = 7) and loss to follow−up before first response assessment (n = 6). A total of 151 individuals were included in analysis.

The enrollment diagram summarizes screening 164 individuals with 13 exclusions throughout the study window (**Figure 1**). A total of 151 patients met the inclusion criteria for this study (**Table 1**). The median age was 48 years (range 29–69), and most patients (84.1%) had squamous cell carcinoma (**Table 1**). FIGO stage distribution ranged from IB (22.5%) to IVA (9.9%), reflecting a broad spectrum of disease severity (**Table 1**). Over half of the cohort (58.9%) presented with tumors ≥4 cm in size, and 38.4% had lymph node metastases at diagnosis (**Table 1**). HPV-16 was the most frequent high-risk subtype (63.6%), followed by HPV-18 (19.9%) and other high-risk variants (16.6%) (**Table 1**).

**Table 1:** Baseline patient characteristics.

Variable	Value
Median Age (Range, years)	48 (29–69)
FIGO stage	
IB	34 (22.5%)
IIA/IIB	55 (36.4%)
IIIA/IIIB	47 (31.1%)
IVA	15 (9.9%)
Histological type	
Squamous Cell Carcinoma	127 (84.1%)
Adenocarcinoma	24 (15.9%)
Tumor size	
<4 cm	62 (41.1%)
≥4 cm	89 (58.9%)
Lymph node metastasis	
Positive	58 (38.4%)
Negative	93 (61.6%)
HPV subtype	
HPV-16	96 (63.6%)
HPV-18	30 (19.9%)
Other High-Risk	25 (16.6%)

At baseline, nearly half of the patients (49.0%) had elevated baseline SCC-Ag levels above 2.0 ng/mL, and 31.8% exhibited CA125 levels exceeding 35 U/mL (**Supplementary Table S1**). Inflammatory markers were also frequently elevated: 37.1% had CRP ≥5.0 mg/L, and 43.0% showed IL-6 ≥6.0 pg/mL (**Supplementary Table S1**). Additionally, 26.5% had an NLR >3.0, while 25.8% presented with a PLR >180, indicating a sizable proportion of patients with an unfavorable inflammatory profile (**Supplementary Table S1**). The 13 excluded patients (9 incomplete baseline biomarkers; 4 lost before first assessment) had stage, histology, tumor size, nodal status, and HPV distributions comparable to the analyzed cohort, suggesting minimal selection bias (**Supplementary Table S2**).

Three months after completing chemoradiotherapy, 70.9% of the cohort achieved a CR, while 16.6% demonstrated a PR (**Table 2**). SD was observed in 7.9% of patients, and 4.6% showed PD (**Table 2**). Overall, 87.4% (CR+PR) responded to treatment, highlighting a generally favorable short-term response rate in this study population (**Table 2**).

**Table 2:** Treatment response at three months post-chemoradiotherapy.

Response	Frequency (%)
Complete Response	107 (70.9%)
Partial Response	25 (16.6%)
Stable Disease	12 (7.9%)
Progressive Disease	7 (4.6%)

In univariate analyses, elevated SCC-Ag, CA125, IL-6, and CRP were all significantly associated with poorer short-term response (**Table 3**). Likewise, high hematologic ratios (NLR >3.0, PLR >180) correlated with lower response rates (**Table 3**).

**Table 3:** Univariate analysis of key baseline biomarkers related to treatment response.

Variable	Cutoff/category	CR/PR (n=132)	SD/PD (n=19)	P-value
SCC-Ag (ng/mL)	>2.0 ng/mL	60 (45.5%)	14 (73.7%)	0.021
CA125 (U/mL)	>35 U/mL	37 (28.0%)	11 (57.9%)	0.007
CRP (mg/L)	≥5.0 mg/L	44 (33.3%)	12 (63.2%)	0.010
IL-6 (pg/mL)	≥6.0 pg/mL	49 (37.1%)	16 (84.2%)	0.002
NLR	>3.0	25 (18.9%)	15 (78.9%)	<0.001
Cumulative HRCTV EQD2 Dose (Gy)	≥85 *vs*. <85	95 (72.0%)	11 (57.9%)	0.161
Overall Treatment Time	≤56 days *vs*. >56 days	100 (75.8%)	10 (52.6%)	0.074
Number of Cisplatin Cycles Completed	≥4 *vs*. <4	90 (68.2%)	12 (63.2%)	0.662

Comparisons of ROC curves indicated that the multi-biomarker panel achieved a higher area under the curve (AUC = 0.86) than any single biomarker alone (**Table 4**). Among individual markers, IL-6, SCC-Ag, and CA125 performed moderately well but none matched the composite panel’s predictive accuracy, underscoring the value of integrating multiple biomarkers (**Table 4**).

**Table 4:** Receiver operating characteristic analysis for predicting CR/PR *vs*. SD/PD.

Predictor	AUC (95% CI)	P-value *vs*. panel	
SCC-Ag	0.72 (0.64–0.78)	<0.001
IL-6	0.74 (0.67–0.80)	<0.001
CA125	0.73 (0.65–0.79)	<0.001
NLR	0.71 (0.63–0.77)	<0.001
Combined Panel	0.86 (0.81–0.90)	–

Using CTCAE v5.0, grade 3–4 toxicities were similar between composite−score risk groups when separated into acute and late windows (**Supplementary Table S3**). For acute events, hematologic grade 3–4 toxicity occurred in 12.7% (8/63) of high−risk *vs* 12.5% (11/88) of low−risk patients (p = 0.97); gastrointestinal 6.3% *vs* 8.0% (p = 0.71); genitourinary 3.2% *vs* 3.4% (p = 0.94); and other 1.6% *vs* 2.3% (p = 0.77). Late grade 3–4 events were infrequent and likewise balanced (hematologic 3.2% *vs* 2.3%, p = 0.73; gastrointestinal 3.2% *vs* 3.4%, p = 0.94; genitourinary 1.6% *vs* 1.1%, p = 0.81; other 1.6% *vs* 1.1%, p = 0.81). Overall, these data corroborate that the composite risk groups did not differ in severe acute or late toxicity.

**Figure 2:**
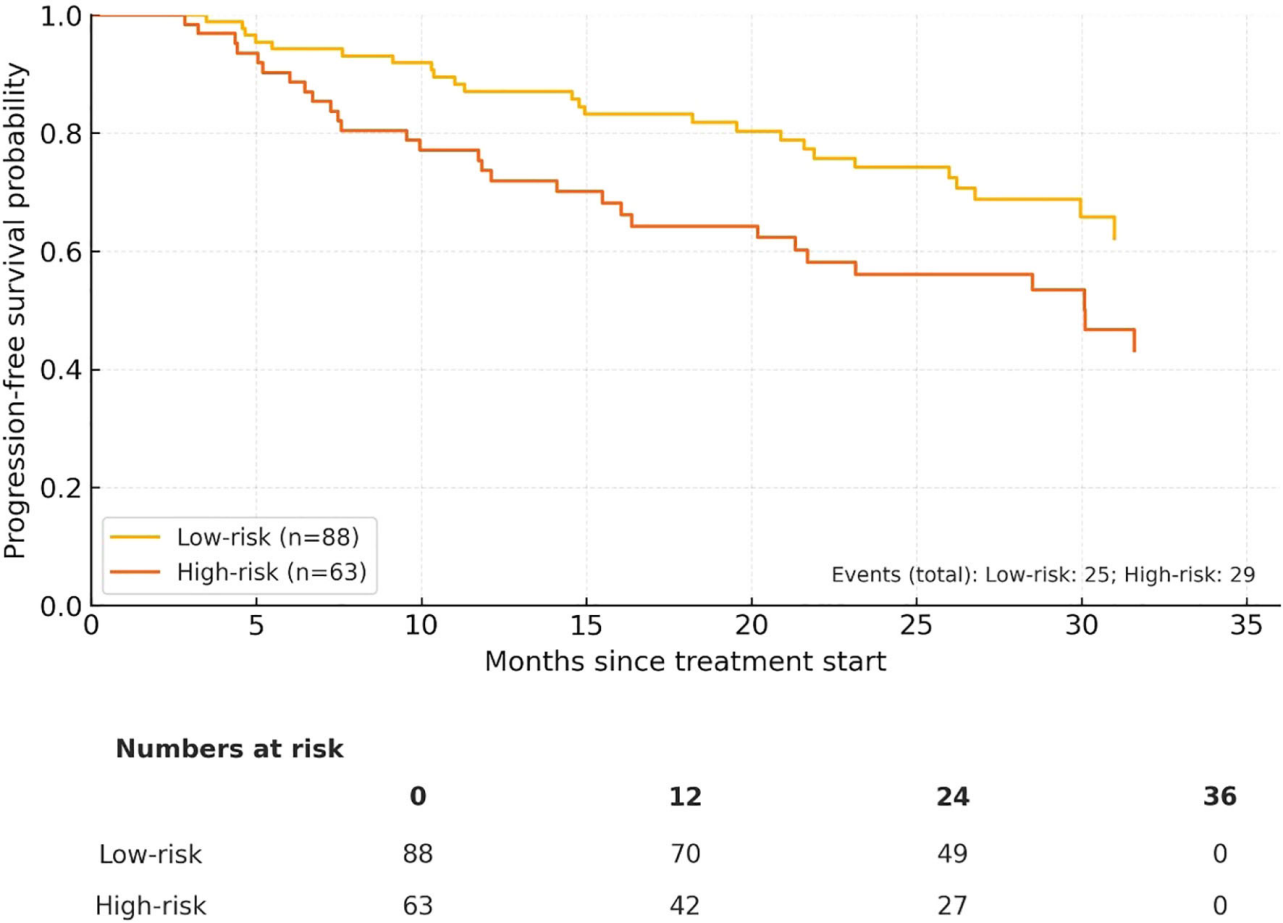
Progression−free survival (PFS) by composite risk group (Low−risk n = 88 *vs* High−risk n = 63). Numbers at risk at 0/12/24/36 months are displayed below the x−axis.

**Figure 3:**
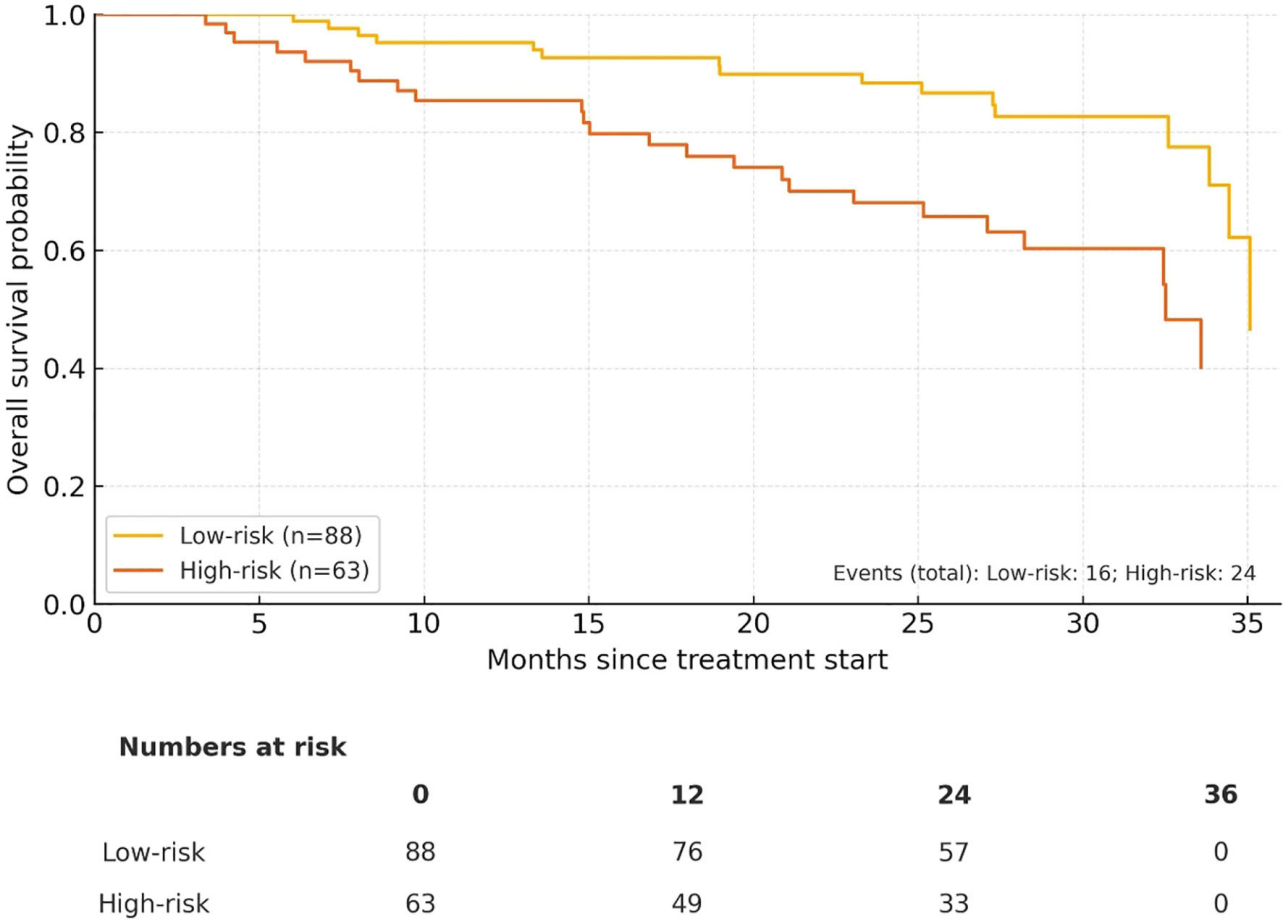
Overall survival (OS) by composite risk group (Low−risk n = 88 *vs* High−risk n = 63). Numbers at risk at 0/12/24/36 months are displayed below the x−axis.

The high-risk cohort had notably poorer survival outcomes. 3−year PFS was 73.8% in the low−risk group *vs* 59.0% in the high−risk group, and 3−year OS was 82.9% *vs* 66.2%, respectively. The total number of events was 54 for PFS (High−risk 29, Low−risk 25) and 40 for OS (High−risk 24, Low−risk 16). Median PFS/OS were not reached within 36 months for either group (**Figures 2**, **3**).

Three−month CR/PR rates declined with advancing stage—91.2% (IB), 90.9% (IIA/IIB), 83.0% (IIIA/IIIB), and 80.0% (IVA)—while PD was uncommon overall but more frequent in advanced disease (0–1.8% in I/II *vs* 6.4–20.0% in III/IVA) (**Supplementary Table S4A**). Within I/II and III/IVA strata, the composite 0–4 score showed similar discrimination for CR/PR (AUC 0.86 and 0.85, respectively), and its prognostic association with PFS/OS persisted in both strata (PFS HR = 1.72 *vs* 1.78; OS HR = 1.80 *vs* 1.89) (**Supplementary Table S4B**). Formal score×stage interaction tests were non−significant for response and survival (all p_interaction ≥ 0.62), indicating performance that is not stage−dependent.

We also assessed the relationship between pathological features and survival. As shown in **Supplementary Table S5** (Progression-Free Survival) and **Supplementary Table S6** (Overall Survival), univariate Cox regression revealed that larger tumor size (≥4 cm), lymph node metastasis, and advanced FIGO stage (III/IVA) were each significantly associated with worse PFS (p < 0.05) and OS (p < 0.05). The composite biomarker score (high-risk *vs*. low-risk) also demonstrated significant prognostic value for both survival endpoints. In contrast, differences in HPV genotype (HPV-16, HPV-18, or other high-risk HPV types) and histological subtype (squamous *vs*. adenocarcinoma) did not reach statistical significance for either PFS or OS (all p > 0.05). After adjusting for relevant clinical covariates in the multivariate models, the composite biomarker score remained an independent predictor of both poorer PFS (HR: 1.75, p = 0.004) and OS (HR: 1.88, p = 0.003). Neither HPV genotype nor histological subtype significantly affected survival outcomes in the adjusted models. We also evaluated cumulative HRCTV EQD2 dose, overall treatment time, and the total number of cisplatin cycles. Although overall treatment time exceeding 56 days showed a near-significant trend toward worse outcomes (p = 0.07 for CR/PR *vs*. SD/PD), it did not remain significant in univariate or multivariable Cox models for either PFS or OS. Similarly, neither cumulative EQD2 dose nor the number of cisplatin cycles predicted survival or treatment response at the p < 0.05 threshold. After adjusting for these factors, our composite biomarker score remained an independent predictor of both PFS (HR: 1.72, p = 0.004) and OS (HR: 1.88, p = 0.003).

By the data cut−off, 54 PFS events and 40 OS events occurred among 151 patients. The primary Cox models used four prespecified covariates (FIGO stage, tumor size, lymph−node status, and the composite score), yielding EPV = 13.5 for PFS and EPV = 10.0 for OS, consistent with commonly cited parsimony thresholds (**Supplementary Table S7**). The composite score remained independently prognostic in these models (PFS HR = 1.75; 95% CI, 1.18–2.67; OS HR = 1.88; 95% CI, 1.23–2.85), with near−identical estimates in a parsimonious three−variable model (PFS HR = 1.73; 95% CI, 1.17–2.58; OS HR = 1.83; 95% CI, 1.21–2.78) (**Supplementary Table S7**). Treatment−delivery factors (EQD2, OTT, cisplatin cycles) were retained transparently and, when added one at a time to the base model, did not materially change the composite score’s association with outcomes, supporting that the models were not overcrowded and the score’s effect is robust to treatment−intensity differences.

Modeling the four panel biomarkers as continuous variables (SCC−Ag, CA125, and IL−6 log2−transformed; NLR per 1−unit increase) yielded AUC = 0.87 (95% CI, 0.82–0.91) for discriminating CR/PR *vs*. SD/PD, which was not different from the dichotomized panel’s performance (AUC = 0.86 [95% CI, 0.81–0.90]; DeLong p = 0.38), indicating that discrimination is not dependent on dichotomization (**Supplementary Table S8**).

For survival, Cox models using the same continuous markers showed Harrell’s C−index = 0.68 (0.62–0.74) for PFS and 0.70 (0.64–0.77) for OS (**Supplementary Table S8**). In a simple chronological split (early = derivation; later = validation), we froze the ≥2 *vs* 0–1 rule and the continuous−marker coefficients from the derivation set and applied them without re−tuning to the validation set: the fixed composite score remained prognostic (PFS HR = 1.68; 95% CI, 1.02–2.77; log−rank p = 0.041; OS HR = 1.82; 95% CI, 1.03–3.21; p = 0.039) and the binary panel retained good short−term response discrimination (AUC = 0.84; 95% CI, 0.76–0.91). Together, these sensitivity analyses support temporal stability of the panel and consistency with the main results.

To assess whether the composite score might proxy treatment intensity, we compared HR−CTV EQD2 (Gy), overall treatment time (OTT, days), and cisplatin cycles between risk groups. Median EQD2 was 87 Gy (83–90) in the low−risk group *vs* 86 Gy (82–89) in the high−risk group (p = 0.29); the proportion achieving EQD2 ≥ 85 Gy was 73.9% (65/88) *vs* 65.1% (41/63), respectively (p = 0.24). Median OTT was 53 days (49–56) *vs* 54 days (50–58) with OTT > 56 days in 25.0% (22/88) *vs* 30.2% (19/63) (p = 0.47). Median cisplatin cycles were 5 (4–6) in both groups, with ≥4 cycles delivered to 68.2% (60/88) *vs* 66.7% (42/63) (p = 0.83). In multivariable analyses that retained these treatment variables and added each one at a time to the base clinical model, the composite score remained independently prognostic for PFS and OS (adjusted HR_PFS = 1.75, HR_OS = 1.88), arguing against the panel acting as a surrogate for treatment intensity (**Supplementary Table S9**).

## Discussion

In this study, a multi-biomarker panel outperformed individual markers in discriminating short-term response and was prognostic for long-term survival among cervical cancer patients undergoing chemoradiotherapy. The composite model achieved a high predictive accuracy for distinguishing responders (CR/PR) from non-responders (SD/PD), exceeding single marker performance. Furthermore, patients categorized as high-risk by the multi-biomarker score demonstrated significantly lower three-year progression-free and overall survival rates compared to those in the low-risk group. These findings highlight the potential clinical value of integrating molecular, inflammatory, and tumor-specific markers into a unified risk-stratification tool for more accurate prognostication and personalized management.

In our analysis of 151 patients with FIGO stage IB–IVA cervical cancer, the multi-biomarker panel significantly enhanced discriminative performance for short-term response (AUC = 0.86) compared to single-marker models in distinguishing responders from non-responders. This outcome aligns with earlier research indicating that SCC-Ag alone correlates with disease-free and overall survival, particularly in early-stage disease (13). Indeed, large-scale studies have validated the benefit of combining tumor markers such as SCC-Ag and p16 with inflammatory markers (e.g., IL-6, CRP) to improve prognostic accuracy (14, 15). Discrepancies among studies may result from variations in ethnicity, staging distributions, and assay methodologies (16, 17), as well as biological heterogeneity in different cervical cancer subtypes and the timing of sample collection (18, 19). These findings highlight the importance of standardized protocols to reliably compare and apply biomarker data across diverse populations.

The observed synergy among SCC-Ag, IL-6, and other markers in our multi-biomarker approach may, in part, be explained by their distinct biological roles. SCC-Ag reflects tumor burden and cell turnover, indicating that elevated levels could represent a more aggressive disease phenotype (20). Inflammatory and immune components also play a key role: IL-6 mediates chronic inflammation and may promote tumor progression and treatment resistance, while CRP levels indicate systemic inflammatory responses (21). Moreover, hematologic ratios such as NLR and PLR may capture the balance between pro-tumor inflammation and anti-tumor immune activity (21). By integrating molecular, immune, and inflammatory signals, the multi-biomarker panel provides a more comprehensive assessment of tumor biology, offering stronger predictive power than single markers alone (22).

We examined tumor-derived markers, inflammatory/immunologic mediators, hematologic indices that summarize systemic inflammation and host immunity, metabolic stress, and viral factors. Biologically, SCC-Ag reflects squamous tumor burden and correlates with recurrence risk, CA125 captures mucin glycoprotein shedding and invasive phenotype, IL-6 drives STAT3-mediated tumor progression and radioresistance, and NLR integrates neutrophil-dominant inflammation versus lymphocyte-mediated anti-tumor immunity in cervical and other solid tumors (8, 13, 17, 21). In our cohort, integrating four prespecified axes—tumor burden, mucin/tumor phenotype, cytokine-driven inflammation, and systemic immune balance yielded superior discrimination for early response and independent prognostic value for survival, with consistent performance across FIGO I/II and III/IVA strata. High-risk patients (score ≥ 2) experienced lower 3-year PFS/OS than low-risk patients, underscoring that complementary biology captured by tumor burden, mucin expression, cytokine signaling, and host inflammatory tone is clinically actionable for CRT risk stratification in locally advanced cervical cancer. Together with prior literature showing SCC-Ag’s prognostic value, the central role of IL-6 and inflammatory indices, and the added value of composite panels over single markers, these findings support adopting a pragmatic, low-cost multi-biomarker approach in routine care while multi-institutional studies refine calibration and generalizability (8, 13, 17, 21).

From a clinical perspective, the refined risk stratification enabled by our composite biomarker score has direct implications for patient management. Similar to how circulating gene panels have guided therapy and justified new patient-specific clinical trials in prostate cancer (23), our findings suggest that distinguishing high- from low-risk cervical cancer patients could inform treatment decisions. Furthermore, emerging methods such as chemogram-based gene expression signatures and organoid drug testing (24, 25) have illustrated the principle of customizing chemotherapy based on biomarker-driven insights. Incorporating dynamic assessments, such as ctDNA kinetics, could further refine the evaluation of treatment response and the timing of therapeutic adjustments (26). High-plex biomarker panels and ongoing trials such as REVISE, which incorporate ctDNA monitoring into chemotherapy decisions, underscore the growing feasibility of translating biomarker-driven approaches into clinical practice (27, 28). Tissue or circulating−tumor–derived assays and MRI/PET−CT–derived quantitative features can capture tumor biology at different scales, but often require specialized platforms, higher cost, and longer turnaround. By contrast, our panel uses widely available clinical assays from a single pre−CRT blood draw, achieved strong discrimination for short−term response and remained prognostic for PFS/OS after adjustment. Going forward, multimodal studies should start from a shared clinical baseline and then add genomic or imaging features on top of the locked four−marker panel to test incremental value and to evaluate fairness across stage, histology, and ethnicity. Such work will clarify when a low−cost panel alone suffices and when genomic/imaging augmentation materially improves clinical utility.

Our baseline four−marker panel (SCC−Ag, CA125, IL−6, and NLR) relies on assays already available in most hospital laboratories: SCC−Ag/CA125 on automated immunoassay analyzers, IL−6 by ELISA, and NLR derived from the routine complete blood count. As such, the incremental direct cost and operational burden are modest, and all analytes can be obtained from a single pre−CRT blood draw. In health systems where MRI/PET−CT availability is limited or costly, a prognostic panel that reliably identifies high−risk patients could support resource prioritization. For example, directing advanced imaging and closer surveillance to high−risk patients while maintaining standard care schedules for low−risk patients. We did not perform a formal cost−effectiveness analysis. Future multi−institutional studies in diverse LMIC settings should evaluate budget impact and cost−effectiveness that incorporate assay procurement, batching/turnaround, and downstream resource use. Standardized pre−analytic procedures and local verification of cut−offs will be essential for scalable and affordable adoption.

The prespecified thresholds used in this study were based on our laboratory reference intervals and common literature values (29). However, IL−6 and CRP immunoassays in particular can show between−platform bias and lot−to−lot variation (30, 31), and CBC−derived indices may differ slightly across hematology analyzers. Before clinical adoption, sites should standardize pre−analytic procedures (specimen type, timing, processing) and analytic calibration (unit traceability, external quality assurance), then verify or recalibrate local cut−offs. For example, by anchoring to the laboratory upper reference limit or by confirming operating points via ROC/Youden against a prespecified endpoint—while preserving the same four−marker structure and the simple ≥2 *vs* 0–1 score rule (29).

This study’s strengths include a prospective design, a relatively large sample size, and a comprehensive multi-biomarker approach incorporating both tumor-specific and inflammatory markers to assess predictive performance and survival outcomes. However, it is also subject to limitations, such as potential selection bias arising from a single-center study in China and the use of specific cut-off values that may vary across different assays or populations. As such, the findings may not be directly generalizable to other populations, particularly those with different ethnic or regional backgrounds. The lack of diversity, potentially limiting the generalizability of the results to other demographic groups. While a baseline four−marker panel offered strong discrimination for early response and was prognostic for PFS/OS, incorporating dynamic biomarkers may further improve risk adaptation. At last, our survival analyses were limited to 36 months, which may underestimate late relapses and late mortality. Extended follow−up and external cohorts with ≥5−year observation will be important to fully characterize late event patterns and confirm long−term prognostic performance. To confirm generalizability across laboratories and populations, the four−marker panel and score rule should be locked and evaluated in retrospective, multi−institutional datasets with stored baseline serum and complete outcomes, and prospective, multicenter cohorts enriched for multi−ethnic representation. Validation should use identical end points (3−month CR/PR *vs* SD/PD; PFS/OS) and report both discrimination (AUC for response; Harrell’s C−index for survival) and calibration (calibration slope/intercept, observed *vs* predicted risk by deciles), with site−level random effects or stratification to account for heterogeneity. Centers should run assays on their local platforms with standardized pre−analytic handling and external quality assurance; cut−offs can be verified locally and, if needed, recalibrated in a secondary analysis while keeping the same four markers and ≥2 *vs* 0–1 decision rule. Clinical utility should be summarized with decision−curve analysis against staging alone, and subgroup performance reported by ethnicity, stage, and histology to assess fairness.

## Conclusion

Our findings indicate that a composite biomarker panel comprising SCC-Ag, CA125, IL-6, and NLR achieves superior predictive performance for both treatment response and survival compared to single-marker models in patients with locally advanced cervical cancer undergoing chemoradiotherapy. In particular, high-risk patients demonstrated significantly lower rates of three-year progression-free and overall survival, underscoring the potential for more intensive treatment or follow-up schedules in this subgroup. By contrast, variation in HPV genotype and histological subtype did not significantly alter prognosis in our cohort, suggesting the composite panel may better capture the multifactorial nature of treatment outcomes.

As immunotherapy becomes integrated into definitive management, the biologic processes captured by our baseline four−marker panel—tumor burden and systemic inflammation—are expected to remain relevant. Thus, its prognostic value is likely to persist in CRT ± immunotherapy settings. Determining whether the panel is treatment−predictive for immunotherapy benefit will require prospective, multi−institutional validation with explicit biomarker×treatment interaction tests in cohorts receiving CRT plus immunotherapy.

## Data Availability

The raw data supporting the conclusions of this article will be made available by the authors, without undue reservation.
