# Supplementary material for: Prognostic value and early response stratification of a multi-biomarker panel in cervical cancer patients undergoing chemoradiotherapy
Source: Front Oncol. 2025 Oct 30;15:1686716. doi: 10.3389/fonc.2025.1686716 (PMC12611648; doi:10.3389/fonc.2025.1686716)
Supplement: Supplementary file 1 [file DataSheet1.docx]

**Supplementary Table S1. Baseline Biomarker Levels and Distribution**

| **Biomarker** | **Cutoff / Reference** | **Median (Range)** | **Above/Below Cutoff (% or n)** |
| --- | --- | --- | --- |
| **SCC-Ag (ng/mL)** | >2.0 ng/mL | 1.9 (0.4–14.2) | Elevated: 74 (49.0%) |
| **CA125 (U/mL)** | >35 U/mL | 27.5 (5.0–135.0) | Elevated: 48 (31.8%) |
| **CRP (mg/L)** | ≥5.0 mg/L | 4.1 (0.7–21.0) | Elevated: 56 (37.1%) |
| **IL-6 (pg/mL)** | ≥6.0 pg/mL | 5.4 (1.3–26.0) | Elevated: 65 (43.0%) |
| **LDH (U/L)** | >245 U/L | 235 (140–390) | Elevated: 47 (31.1%) |
| **NLR** | >3.0 | 2.8 (1.0–7.5) | High: 40 (26.5%) |
| **PLR** | >180 | 170 (80–340) | High: 39 (25.8%) |
| **LMR** | <3.0 | 3.3 (1.2–6.4) | Low: 32 (21.2%) |

### ****Supplementary Table S2. Baseline characteristics of excluded patients vs. analysis cohort****

| **Characteristic** | **Excluded (n = 13)** | **Analyzed (n = 151)** | **p‑value** |
| --- | --- | --- | --- |
| **Age (years),** median (IQR) | **49 (41–57)** | **48 (42–56)** | — |
| **FIGO stage**, n (%) |  |  | **0.994** |
| IB | **3 (23.1%)** | **34 (22.5%)** |  |
| IIA/IIB | **5 (38.5%)** | **55 (36.4%)** |  |
| IIIA/IIIB | **4 (30.8%)** | **47 (31.1%)** |  |
| IVA | **1 (7.7%)** | **15 (9.9%)** |  |
| **Histology**, n (%) |  |  | **1.000** |
| Squamous cell carcinoma | **11 (84.6%)** | **127 (84.1%)** |  |
| Adenocarcinoma | **2 (15.4%)** | **24 (15.9%)** |  |
| **Tumor size ≥4 cm**, n (%) | **8 (61.5%)** | **89 (58.9%)** | **1.000** |
| **Lymph‑node metastasis (positive)**, n (%) | **5 (38.5%)** | **58 (38.4%)** | **1.000** |
| **HPV genotype**, n (%) |  |  | **0.961** |
| HPV‑16 | **8 (61.5%)** | **96 (63.6%)** |  |
| HPV‑18 | **3 (23.1%)** | **30 (19.9%)** |  |
| Other high‑risk | **2 (15.4%)** | **25 (16.6%)** |  |
| **Reason for exclusion**, n |  |  | — |
| Incomplete baseline biomarkers | **9** | — |  |
| Lost to follow‑up before 3‑month assessment | **4** | — |  |

### ****Supplementary Table S3. Grade 3–4 treatment‑related toxicities****

| **Toxicity category** | **Acute (start of EBRT to 90 days after completion of all RT)** | | | **Late (>90 days after completion of all RT)** | | |
| --- | --- | --- | --- | --- | --- | --- |
|  | **High‑risk**  **(n=63)** | **Low‑risk**  **(n=88)** | **p (acute)** | **High‑risk (n=63)** | **Low‑risk (n=88)** | **p (late)** |
| **Hematologic, G3–4** | **8 (12.7%)** | **11 (12.5%)** | **0.97** | **2 (3.2%)** | **2 (2.3%)** | **0.73** |
| **Gastrointestinal, G3–4** | **4 (6.3%)** | **7 (8.0%)** | **0.71** | **2 (3.2%)** | **3 (3.4%)** | **0.94** |
| **Genitourinary, G3–4** | **2 (3.2%)** | **3 (3.4%)** | **0.94** | **1 (1.6%)** | **1 (1.1%)** | **0.81** |
| **Other, G3–4** | **1 (1.6%)** | **2 (2.3%)** | **0.77** | **1 (1.6%)** | **1 (1.1%)** | **0.81** |

## ****Supplementary Table S4. Stage‑wise short‑term response and stage‑stratified performance****

| **A. Short‑term response (3 months) by FIGO stage** | | | | |
| --- | --- | --- | --- | --- |
| **FIGO stage** | **N** | **CR/PR, n (%)** | **SD, n (%)** | **PD, n (%)** |
| **IB** | 34 | **31 (91.2%)** | 3 (8.8%) | **0 (0%)** |
| **IIA/IIB** | 55 | **50 (90.9%)** | 4 (7.3%) | **1 (1.8%)** |
| **IIIA/IIIB** | 47 | **39 (83.0%)** | 5 (10.6%) | **3 (6.4%)** |
| **IVA** | 15 | **12 (80.0%)** | 0 (0%) | **3 (20.0%)** |
| **Total** | **151** | **132 (87.4%)** | **12 (7.9%)** | **7 (4.6%)** |
| **B. Early (I/II) vs Advanced (III/IVA) strata** | | | | |
| **Stratum** | **N** | **Panel (0–4 score) AUC for CR/PR (95% CI)** | **PFS HR (High vs Low; 95% CI)** | **OS HR (High vs Low; 95% CI)** |
| **I/II** | 89 | **0.86 (0.79–0.92)** | **1.72 (1.03–2.85)** | **1.80 (1.04–3.10)** |
| **III/IVA** | 62 | **0.85 (0.77–0.91)** | **1.78 (1.02–3.07)** | **1.89 (1.05–3.40)** |

***Score×stage interaction (global tests)*:**

**Response (logistic):** p_interaction**= 0.62**

**PFS (Cox):** p_interaction**= 0.88**

**OS (Cox):** p_interaction**= 0.84**

**Supplementary Table S5. Cox Regression Analyses for HPV and Pathological Variables on PFS**

| **Variable** | **Subcategory** | **Univariate Analysis, HR (95% CI)** | **p-Value** | **Multivariate Analysis, HR (95% CI)** | **p-Value** |
| --- | --- | --- | --- | --- | --- |
| **HPV Genotype** | HPV-16 vs. HPV-18 | 1.20 (0.75–1.92) | 0.45 | 1.18 (0.70–1.86) | 0.48 |
|  | HPV-16 vs. Other High-Risk | 1.35 (0.82–2.02) | 0.19 | 1.33 (0.80–2.03) | 0.21 |
|  | HPV-18 vs. Other High-Risk | 1.10 (0.70–1.65) | 0.65 | 1.06 (0.66–1.62) | 0.77 |
| **Histological Type** | Squamous vs. Adenocarcinoma | 1.28 (0.89–1.91) | 0.16 | 1.22 (0.85–1.92) | 0.21 |
| **Tumor Size** | <4 cm vs. ≥4 cm | 1.58 (1.06–2.36) | 0.02 | 1.45 (1.01–2.19) | 0.04 |
| **Lymph Node Metastasis** | Negative vs. Positive | 1.72 (1.08–2.45) | 0.03 | 1.65 (1.04–2.45) | 0.04 |
| **FIGO Stage** | I/II vs. III/IVA | 1.90 (1.20–2.90) | 0.004 | 1.78 (1.10–2.76) | 0.01 |
| **Composite Biomarker Score** | <2 vs. ≥2 (High-Risk) | 1.82 (1.24–2.72) | 0.002 | 1.75 (1.18–2.67) | 0.004 |
| **Cumulative HRCTV EQD2 Dose (Gy)** | ≥85 vs. <85 | 1.30 (0.84–2.01) | 0.25 | 1.37 (0.90–2.08) | 0.14 |
| **Overall Treatment Time** | ≤56 days vs. >56 days | 1.45 (0.93–2.21) | 0.10 | 1.27 (0.81–1.93) | 0.28 |
| **Number of Cisplatin Cycles Completed** | ≥4 vs. <4 | 1.20 (0.77–1.87) | 0.41 | 1.18 (0.75–1.85) | 0.47 |

**Supplementary Table S6. Cox Regression Analyses for HPV and Pathological Variables on OS**

| **Variable** | **Subcategory** | **Univariate Analysis, HR (95% CI)** | **p-Value** | **Multivariate Analysis, HR (95% CI)** | **p-Value** |
| --- | --- | --- | --- | --- | --- |
| **HPV Genotype** | HPV-16 vs. HPV-18 | 1.22 (0.78–1.96) | 0.42 | 1.15 (0.69–1.88) | 0.52 |
|  | HPV-16 vs. Other High-Risk | 1.40 (0.89–2.10) | 0.13 | 1.36 (0.84–2.05) | 0.18 |
|  | HPV-18 vs. Other High-Risk | 1.08 (0.68–1.66) | 0.71 | 1.05 (0.64–1.65) | 0.81 |
| **Histological Type** | Squamous vs. Adenocarcinoma | 1.35 (0.92–2.02) | 0.14 | 1.27 (0.86–1.99) | 0.18 |
| **Tumor Size** | <4 cm vs. ≥4 cm | 1.67 (1.11–2.43) | 0.01 | 1.59 (1.07–2.38) | 0.02 |
| **Lymph Node Metastasis** | Negative vs. Positive | 1.80 (1.10–2.58) | 0.02 | 1.72 (1.05–2.53) | 0.03 |
| **FIGO Stage** | I/II vs. III/IVA | 1.95 (1.23–3.02) | 0.003 | 1.88 (1.15–2.96) | 0.009 |
| **Composite Biomarker Score** | <2 vs. ≥2 (High-Risk) | 1.95 (1.29–2.94) | 0.001 | 1.88 (1.23–2.85) | 0.003 |
| **Cumulative HRCTV EQD2 Dose (Gy)** | ≥85 vs. <85 | 1.35 (0.86–2.05) | 0.22 | 1.32 (0.85–2.02) | 0.24 |
| **Overall Treatment Time** | ≤56 days vs. >56 days | 1.53 (0.96–2.32) | 0.08 | 1.45 (0.90–2.30) | 0.11 |
| **Number of Cisplatin Cycles Completed** | ≥4 vs. <4 | 1.15 (0.71–1.79) | 0.54 | 1.12 (0.70–1.79) | 0.63 |

### ****Supplementary Table S7. Observed events and multivariable model parsimony****

| **Endpoint** | **N** | **Events (n)** | **Primary covariates in base model (k)** | **EPV (events/k)** | **Composite score (adj. HR, 95% CI)*** | **Parsimonious model† (adj. HR, 95% CI)** |
| --- | --- | --- | --- | --- | --- | --- |
| **PFS** | 151 | **54** | 4 | **13.5** | **1.75 (1.18–2.67)** | **1.73 (1.17–2.58)** |
| **OS** | 151 | **40** | 4 | **10.0** | **1.88 (1.23–2.85)** | **1.83 (1.21–2.78)** |

^*^Adjusted HR shown for **high‑risk (score ≥ 2) vs low‑risk (score 0–1)** in the **base multivariable Cox model**.

^†^Parsimonious model includes **FIGO stage** (III/IVA vs I/II), **lymph‑node status** (positive vs negative), and the **composite score** (≥2 vs 0–1); tumor size is omitted to illustrate model parsimony. Treatment‑delivery variables (**EQD2, OTT, cisplatin cycles**) are **kept visible** and were evaluated by **adding each singly** to the base model. EPV = events‑per‑variable.

### ****Supplementary Table S8. Sensitivity analyses****

| **Analysis** | **Endpoint** | **Estimate (95% CI)** |
| --- | --- | --- |
| **Continuous markers (logistic)**^†^ | CR/PR vs. SD/PD | **0.87 (0.82–0.91)** |
| **Dichotomized panel (≥2 vs 0–1)** | CR/PR vs. SD/PD | **0.86 (0.81–0.90)** |
| **DeLong test** | — | **0.38** |
| **Continuous markers (Cox)**^†^ | PFS | **0.68 (0.62–0.74)** |
| **Continuous markers (Cox)**^†^ | OS | **0.70 (0.64–0.77)** |
| **Fixed score (≥2 vs 0–1)**^‡^ | PFS | **1.68 (1.02–2.77)**; log‑rank **p = 0.041** |
| **Fixed score (≥2 vs 0–1)**^‡^ | OS | **1.82 (1.03–3.21)**; log‑rank **p = 0.039** |
| **Fixed panel (binary)**^‡^ | CR/PR vs. SD/PD | **0.84 (0.76–0.91)** |

^†^Continuous‑marker models: SCC‑Ag, CA125, and IL‑6 were **log2‑transformed, NLR** modeled per 1‑unit increase. Logistic regression (CR/PR vs SD/PD) AUC **95% CIs were calculated by DeLong**. Cox models (PFS/OS) **C‑index 95% CIs were calculated by bootstrap**.

^‡^Chronological split: cohort divided at the **median enrollment date** (early = derivation; late = validation). The **score threshold (≥2)** and the continuous‑marker model **coefficients were frozen from the derivation set** and applied to the validation set **without re‑tuning**. Validation “Fixed panel (binary)” AUC is for the **≥2 vs 0–1** classifier.

### ****Supplementary Table S9. Treatment‑delivery characteristics by composite risk group****

| **Variable** | **Low‑risk (score 0–1, n = 88)** | **High‑risk (score ≥2, n = 63)** | **p‑value** |
| --- | --- | --- | --- |
| **Cumulative HR‑CTV EQD2 (Gy), median [IQR]** | **87** [83–90] | **86** [82–89] | 0.29 |
| **EQD2 ≥85 Gy, n/N (%)** | **65/88 (73.9%)** | **41/63 (65.1%)** | 0.24 |
| **Overall treatment time (days), median [IQR]** | **53** [49–56] | **54** [50–58] | 0.18 |
| **OTT >56 days, n/N (%)** | **22/88 (25.0%)** | **19/63 (30.2%)** | 0.47 |
| **Cisplatin cycles, median [IQR]** | **5** [4–6] | **5** [4–6] | 0.75 |
| **Cycles ≥4, n/N (%)** | **60/88 (68.2%)** | **42/63 (66.7%)** | 0.83 |
